# Efficacy of acupuncture for pain relief in patients receiving extracorporeal shock wave lithotripsy: a meta-analysis of randomized controlled studies

**DOI:** 10.3389/fmed.2023.1114485

**Published:** 2023-06-02

**Authors:** Hsiao-Tien Chen, Kuo-Chuan Hung, Yao-Chin Hsu, Jinn-Rung Kuo, Ying-Jen Chang, I-Wen Chen, Cheuk-Kwan Sun

**Affiliations:** ^1^Department of Chinese Medicine, Chi Mei Medical Center, Tainan, Taiwan; ^2^Department of Anesthesiology, Chi Mei Medical Center, Tainan, Taiwan; ^3^Department of Neurosurgery, Chi Mei Medical Center, Tainan, Taiwan; ^4^Department of Medical Research, Chi Mei Medical Center, Tainan, Taiwan; ^5^Department of Recreation and Health-Care Management, College of Recreation and Health Management, Chia Nan University of Pharmacy and Science, Tainan, Taiwan; ^6^Department of Anesthesiology, Chi Mei Medical Center, Liouying, Tainan, Taiwan; ^7^Department of Emergency Medicine, E-Da Hospital, I-Shou University, Kaohsiung City, Taiwan; ^8^School of Medicine for International Students, College of Medicine, I-Shou University, Kaohsiung City, Taiwan

**Keywords:** acupuncture, extracorporeal shock wave lithotripsy, meta-analysis, pain, urolithiasis

## Abstract

**Background:**

This meta-analysis aimed at investigating the efficacy of acupuncture for pain relief in patients receiving extracorporeal shock wave lithotripsy (ESWL).

**Methods:**

Randomized controlled trials comparing the efficacy of acupuncture with conventional treatments were retrieved from major electronic databases (e.g., MEDLINE, EMBASE, and Cochrane Library) until August 28, 2022. The primary outcome was the response rate (i.e., rate of pain relief), while secondary outcomes included stone-free rate, satisfaction rate, duration of ESWL, peri-/post-procedural pain score, and risk of adverse events.

**Results:**

Thirteen eligible studies involving 1,220 participants published between 1993 and 2022 were analyzed. Pooled results indicated that acupuncture had a better response rate compared to conventional treatments (RR = 1.17, 95% CI: 1.06–1.3, *p* = 0.003, seven trials, *n* = 832). Despite no difference in ESWL duration (MD = 0.02 min, 95% CI: −1.53 to 1.57, *p* = 0.98, three trials, *n* = 141), stone-free rate (RR = 1.11, 95% CI: 1–1.25, *p* = 0.06, six trials, *n* = 498), and satisfaction rate (RR = 1.51, 95% CI: 0.92–2.47, *p* = 0.1, three trials, *n* = 334) between the two groups, the acupuncture group had a lower risk of adverse events (RR = 0.51, 95% CI: 0.33–0.79, *p* = 0.003, five trials, *n* = 327), peri- (MD = −1.91 points, 94% CI: −3.53 to −0.28, *p* = 0.02, four trials, *n* = 258 patient) and post-procedural (MD = −1.07, 95% CI: −1.77 to −0.36, *p* = 0.003, four trials, *n* = 335) pain score.

**Conclusion:**

The results of this meta-analysis showed that the use of acupuncture in patients receiving ESWL was associated with a higher pain relief rate and a lower risk of adverse events, suggesting feasibility of its use in this clinical setting.

**Systematic review registration:**

https://www.crd.york.ac.uk/prospero/, identifier: CRD42022356327.

## 1. Introduction

Urolithiasis, which is one of the most prevalent diseases of the urinary system that affects more than 12% of the global population (1, 2), usually manifests with pain, urinary tract infections, and even hydronephrosis when the ureter is obstructed (3). In contrast to open nephrotomy, minimally invasive strategies such as extracorporeal shock wave lithotripsy (ESWL), ureteroscopic lithotripsy (URSL), percutaneous nephrolithotripsy (PCNL), and retroperitoneal ureterolithotripsy (RPUL) are the mainstream treatments in current clinical practice (3, 4). Extracorporeal shock wave lithotripsy (ESWL), which harnesses high-energy shock waves and pressure for stone fragmentation (5), has been described as an epoch-making therapeutic modality because of its simplicity, noninvasiveness (6), effectiveness, and low morbidity (7) especially for renal stones smaller than 2 cm in diameter or those located at the upper ureter (8). Nevertheless, the higher the energy used in ESWL, the more severe the adverse side-effects (9). Besides, patients may experience pain or anxiety (10) because of hematuria, stones retention and urinary tract infection after the procedure (11).

The sources of pain during ESWL can be two-folded. While somatic pain may be caused by shock waves penetrating through the superficial body structures (i.e., skin and muscle) (12), visceral pain can result from shock waves that reach the deeper structures (e.g., ribs, nerves, and the kidney capsule) (13). Although local anesthetics, non-steroidal anti-inflammatory drugs (NSAIDs), and opioids are the common analgesics being used during ESWL in daily practice (14), no consensus has been reached on the optimal analgesic regimen. Acupuncture has long been used as a mode of analgesia (15) that improves lower urinary tract symptoms (16, 17) through inducing ureteral smooth muscle relaxation (18), thereby achieving renal colic pain relief (19). Previous findings have shown that the use of acupuncture during ESWL could reduce analgesic requirement, alleviate anxiety (20–22), and improve the success rate of the procedure (23). Despite growing evidence in support of acupuncture as an analgesic measure during lithotripsy, the efficacy has not been systematically scrutinized. The aim of the current meta-analysis was to examine the role of acupuncture in lithotripsy through systematically reviewing currently available literature based on a comparison of treatment response rate, change in pain severity, stone-free rate, and satisfaction rate between patients receiving acupuncture and those undergoing other analgesic strategies during ESWL.

## 2. Methods

The registered protocol for this systematic review and meta-analysis can be accessed at https://www.crd.york.ac.uk/prospero/, number: CRD42022356327. The research was conducted based on the Preferred Reporting Items for Systematic Reviews and Meta-Analyses Statement (PRISMA) guidelines.

### 2.1. Information sources and search strategy

Literature search was performed to identify eligible randomized controlled trials (RCTs) investigating the effectiveness of acupuncture for pain relief in patients receiving ESWL. Major databases, namely MEDLINE, Embase, and Cochrane CENTRAL register of controlled trials, were searched from their inception dates till August 28, 2022. The key words and medical subject headings (e.g., MeSH terms in Medline) that were used for searching included: (“extracorporeal shock wave lithotripsy” or “shock wave lithotripsy” or “urolithiasis” or “nephrolithiasis” or “Ureteral calculi”) and (“Acupuncture” or “Electro acupuncture” or “Laser acupuncture” or “Needle acupuncture” or “auricular acupuncture” or “manual acupuncture” or “acupoint injection” or “acupoint treatment”). In addition, we also searched the Google scholar, the China National Knowledge Infrastructure (CNKI) database, VIP Information Database, Chinese Biomedical Literature Database (CBM), and Wanfang Database to avoid omitting potentially eligible studies. The reference lists of the published systematic reviews and all of the included RCTs were also screened to identify relevant studies. We did not place restrictions on language, sample size, publication date, and country of publication. The search strategies for one of these databases (i.e., Medline) is provided in Supplementary Table 1.

### 2.2. Inclusion and exclusion criteria

Inclusion criteria were: (a) Patient population: Adult participants (≥18 years) receiving ESWL; (b) Intervention: Acupuncture therapies regardless of its location or type; (c) Comparison: Usual care (e.g., NSAIDS, opioids) or sham acupuncture; (d) Outcomes: Total response rate, treatment-related complications, pain score, and other procedure-related outcomes. Exclusion criteria included: (1) studies in which ESWL was not performed; (2) those that did not use acupuncture as the main therapeutic method; and (3) those that combined acupuncture with other conventional interventional strategies (e.g., NSAIDS, opioids).

### 2.3. Selection process and data collection

Two independent reviewers independently screened the literature for potentially eligible articles in accordance with the inclusion and exclusion criteria. A full text review of the selected trials was conducted to determine their eligibility. Disagreements were resolved through consulting with a third author. As part of the data extraction process, the two independent reviewers carried out separate data extraction processes that involved information pertinent to author information (e.g., first author), characteristics of participants (e.g., gender distribution), sample size, intervention details, drugs for conventional intervention, and country of origin.

### 2.4. Outcomes and definitions of data items

This study was designed to investigate the response rate, which referred to the percentage of participants experiencing effective pain relief after intervention (i.e., primary outcome). The definition of effective pain relief was based on that of each study. The secondary outcome included stone-free rate, risks of adverse events based on the definition of individual studies regardless of the timing of occurrence, satisfaction rate, duration of ESWL, hemodynamic profile (i.e., systolic and diastolic blood pressure, heart rate) during ESWL, as well as peri- and post-procedural pain score. For the current study, acupuncture encompassed all procedures involving the technique of acupuncture, namely conventional acupuncture, auricular acupuncture, laser acupuncture, electroacupuncture, acupuncture injection, and acupoint electrical stimulation.

### 2.5. Risks of bias assessment

Two independent reviewers appraised the methodological qualities of each included RCT according to the Cochrane risk-of-bias tool for RCTs (RoB 2.0), which comprises five domains for assessment, namely selection of the reported results, deviations from intended interventions, outcome measurement, randomization process, and missing outcome (24). The RoB categorized the risk in each domain into three categories: low risk, unclear risk, and high risk. Whenever there was a disagreement between the two reviewers, a third reviewer was consulted until consensus was reached.

### 2.6. Evidence quality assessment

Two independent reviewers evaluated the degree of certainty of the evidence that was categorized into high, moderate, low, and very low through assessing the probability of study limitations, publication bias, effect consistency, imprecision, and indirectness according to GRADE (grading of recommendations assessment, development and evaluation) guidance. All disagreements regarding ratings were resolved through discussion.

### 2.7. Data analysis

All data analyses were performed with the Review Manager (RevMan) version 5.4.1 software (The Nordic Cochrane Center, The Cochrane Collaboration, Copenhagen, Denmark). Continuous variables that were assessed with the same scale are expressed as mean difference (MD) with a 95% confidence interval (CI), while other data are presented as standardized mean difference (SMD). For a dichotomous variable (i.e., efficacy and complication rate), the effect size is expressed as risk ratio (RR) with 95% confidence interval (CI). Overlapping in sample size assessment in studies with more than two intervention arms was prevented by dividing participants in the control group into separate subgroups to compare with their counterparts in the corresponding specific treatment arm as previously described (25). While the means and SDs were preserved in spite of division of the total number of participants on encountering continuous variables, both the event number and the total number of participants were divided for categorical outcomes. In view of the heterogeneity of the clinical and population parameters involved, the Mantel–Haenszel random effects model was chosen for outcome analysis. The degree of heterogeneity was considered significant when *I*^2^ statistics was >50% (26) where a leave-one-out sensitivity analysis was conducted to test robustness of the result. A funnel plot was examined to detect the possibility of publication bias when at least 10 trials were involved. A *p*-value of < 0.05 was deemed statistically significant. The statistical approach adopted in the present meta-analysis was as previously described (27–29).

## 3. Results

### 3.1. Selection, characteristics, and quality of studies

**Figure 1:**
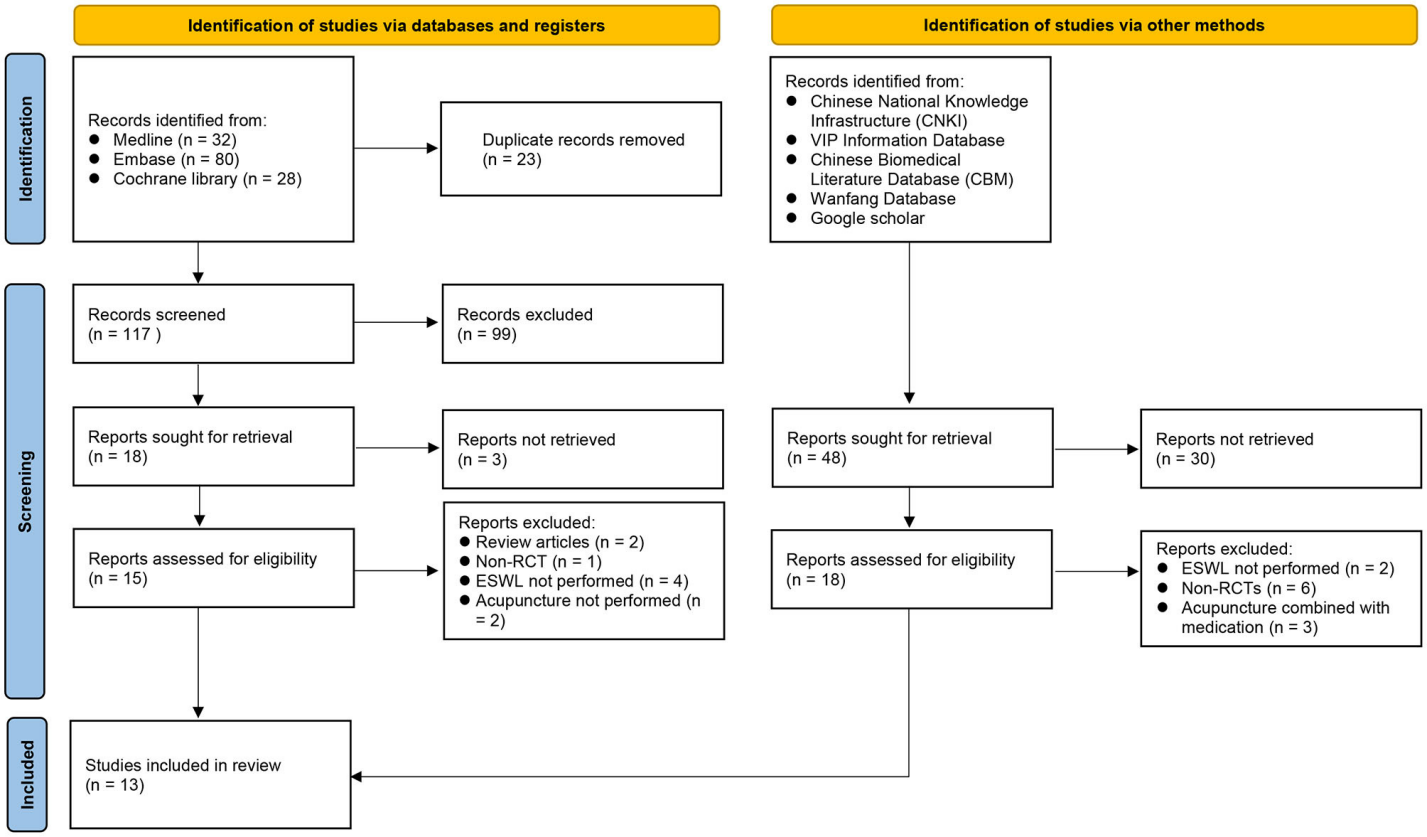
Flow diagram of study selection for the current meta-analysis.

Of the 140 records retrieved from the three main databases, 23 were removed because of duplications. Of the remaining 117 records, 99 were deemed ineligible based on their titles and abstracts. After a further exclusion of nine reports after a full-text screening of the remaining 15 trials, six RCTs were considered eligible for inclusion in the current study (Figure 1). Moreover, seven additional RCTs were identified through the examination of other databases (e., CNKI). In total, 13 RCTs being conducted in China (*n* = 7) (30–36), Taiwan (*n* = 1) (23), Iran (*n* = 1) (37), Turkey (*n* = 1) (38), Germany (*n* = 1) (20), Italy (*n* = 1) (21), and America (*n* = 1) (22) published from 1993 to 2022 were included.

The characteristics of the included studies are summarized in Table 1. The 13 studies included 1,220 patients, of whom 613 were in the acupuncture group, and 607 belonged to the control group. The proportion of male gender ranged from 35 to 79%. The age of participants ranged from 35 to77 years. In the acupuncture group, three types of acupuncture techniques were involved: acupuncture (seven trials) (20, 30, 31, 33–36), electroacupuncture (four trials) (22, 23, 37, 38), electroacupuncture combine with auricular acupressure (one trial) (21), and acupuncture injection (one trial) (32). One study randomized the patients into three groups, namely two intervention groups (i.e., acupuncture and electroacupuncture) and one control group (i.e., dolantin) (30). The details for acupuncture technique are demonstrated in Table 2. In terms of controls, opioids (*n* = 6) (20, 23, 30, 31, 37, 38), sham acupuncture (*n* = 2) (21, 22), no intervention (*n* = 4) (32–34, 36), and racemic anisodamine hydrochloride (*n* = 1) (35) were adopted.

**Table 1:** Characteristics of studies (*n* =13).

**References**	**Age (years)^a^**	***N* ^a^**	**Male (%)**	**Stone diameter (mm)**	**Intervention/control groups**	**Country**
Wang et al. (30)	39 vs. 38	40 vs. 20	62	5–45 vs. 5–18	Acupuncture/dolantin	China
Agah and Falihi (37)	52 vs. 52	50 vs. 50	60	16 vs.18	Electroacupuncture/morphine, diazepam	Iran
Resim et al. (38)	35 vs. 43	17 vs. 18	57	12.4 vs.12.3	Electroacupuncture/tramadol, midazolam	Turkey
Hodzic et al. (20)	56 vs. 57	86 vs. 78	53	8.1 vs.7.5	Acupuncture/dolantin, diazepam	Germany
Wang et al. (22)	46 vs. 44	29 vs. 27	39	11 vs.10	Electroacupuncture/sham acupuncture	America
Mora et al. (21)	76 vs. 77	50 vs. 50	35	NA	Auricular acupressure and electroacupuncture/sham acupuncture	Italy
Hou (31)	48 vs. 48	80 vs. 80	57	21 vs. 21	Acupuncture/dolantin, tramadol	China
Chen et al. (23)	44 vs. 48	25 vs. 49	79	7.5 vs. 6.7	Electroacupuncture/opium analgesic	Taiwan
Zhang et al. (32)	39 vs. 37	32 vs. 32	68	NA	Acupuncture injection/no medication	China
Pei (33)	42 vs. 43	31 vs. 31	50	NA	Acupuncture/no medication	China
Wang (34)	37 vs. 35	60 vs. 60	62	14.3 vs.14.5	Acupuncture/no medication	China
Yu et al. (35)	40 vs. 39	30 vs. 29	71	8.4 vs. 8.3	Acupuncture/racemic anisodamine hydrochloride	China
Cheng et al. (36)	49 vs. 50	83 vs. 83	64	NA	Acupuncture/no medication	China

^a^Present as intervention vs control groups.

NA, not available.

**Table 2:** Detail of acupuncture (*n* = 13).

**References**	**Acupoint**	**Depth of insertion**	**Acupoint stimulation**	**Retention time (min)**
Wang et al. (30)	He Gu point San Yin Jiao	1.7–3.3 cm	Twist and turn in conventical acupuncture The sparse and dense waves are used for electroacupuncture	15–20 (before ESWL)
Agah and Falihi (37)	ST 36 UB 60	0.30 × 18 mm	The low voltage-high frequency method	30 (before ESWL)
Resim et al. (38)	Bladder points 20, 21, 22, 23, and 52 Ear points shenmen, kidney, and bladder	3–4 cm	Felt the sensation of De qi as numbness	30 (before ESWL)
Hodzic et al. (20)	He Gu point	0.20 × 15mm	NA	0–21 (during ESWL)
Wang et al. (22)	Auricular acupuncture Four gate acupoint	0.25 × 20 mm	NA	30 (during ESWL)
Mora et al. (21)	Auricular acupuncture	1mm	NA	Until the patient was delivered to the hospital
Hou (31)	Kidney Yu, Bladder Yu San Yin Jiao, ST 36	NA	NA	During ESWL
Chen et al. (23)	BL-40	3 cm	Felt the sensation of De qi as numbness	20 (before ESWL)
Zhang et al. (32)	Kidney Yu	NA	Felt the sensation of De qi as numbness	25–30 (before ESWL)
Pei (33)	Kidney Yu, San Yin Jiao, ST 36	0.3 × 40 mm	Felt the sensation of De qi as numbness	30 (after ESWL)
Wang (34)	Auricular acupuncture Kidney Yu, ST 36	3 cm	Felt the sensation of De qi as numbness	30 (after ESWL)
Yu et al. (35)	Kidney Yu, Bladder Yu, Yin Ling Quan, Qi Hai, Guan Yuan, San Yin Jiao, Tai Chong, He Gu	0.2 × 5 mm	Felt the sensation of De qi as numbness	10 (before ESWL)
Cheng et al. (36)	Zhi Men, Kidney Yu, Ji Shen, San Jiao Yu, Bladder Yu, Jing Men, Zhi Mou, Wei Yang, Yin Ling Quan	3 cm	Felt the sensation of De qi as numbness	30 (after ESWL)

NA, not available; ESWL, extracorporeal shock wave lithotripsy.

Of the five studies (23, 30, 33, 34, 38) that reported information about adverse events, only one (30) mentioned three cases of nausea and two cases of palpitations in the acupuncture analgesia group. Two other trials (34) identified poor stone evacuation after ESWL as the underlying cause of adverse complications. Two studies (23, 38) noted that none of the patients receiving acupuncture experienced adverse reactions.

**Figure 2:**
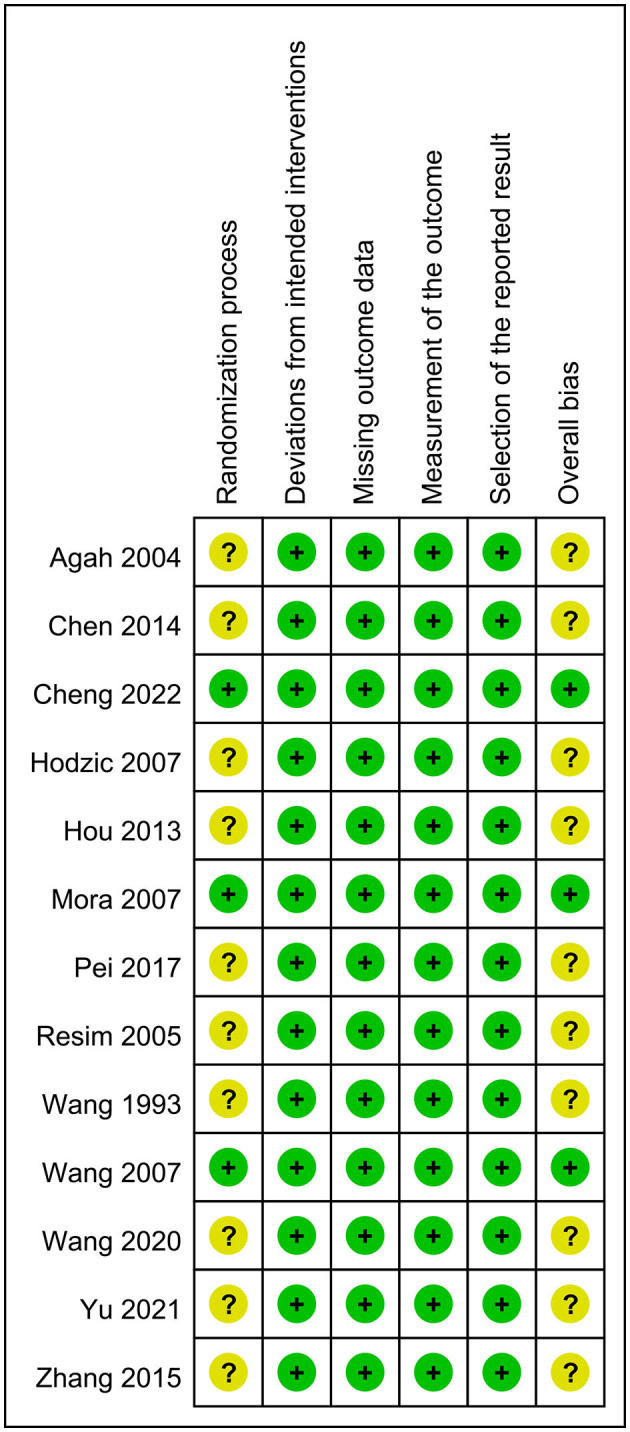
Summary of risks of bias. Green: low risk; yellow: some concerns.

The results of risk of bias assessment are summarized in Figure 2. The rating of “having some concerns” was given to the risk of bias for the randomization process in ten trials that failed to disclose details about allocation sequence concealment. The overall bias was graded as “low” and “having some concerns” in three and 10 studies, respectively.

### 3.2. Results of syntheses

**Figure 3:**
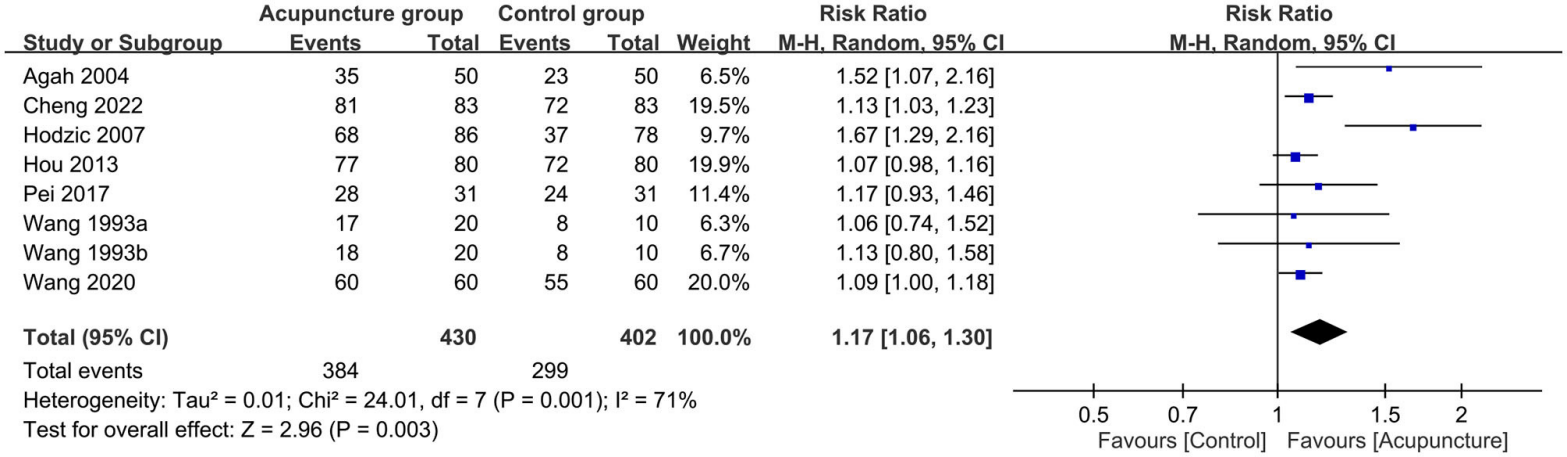
Forest plot comparing response rate between acupuncture and control groups. RR, risk ratio; M-H, Mantel–Haenszel; CI, confidence interval.

**Figure 4:**
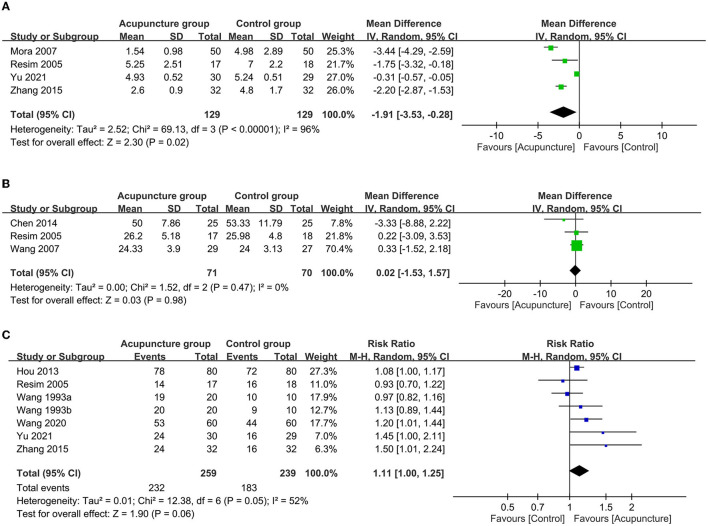
Forest plot comparing the **(A)** peri-procedural pain score; **(B)** duration of extracorporeal shock wave lithotripsy (ESWL); and **(C)** stone-free rate between acupuncture and control groups. CI, confidence interval; IV, inverse variance.

**Figure 5:**
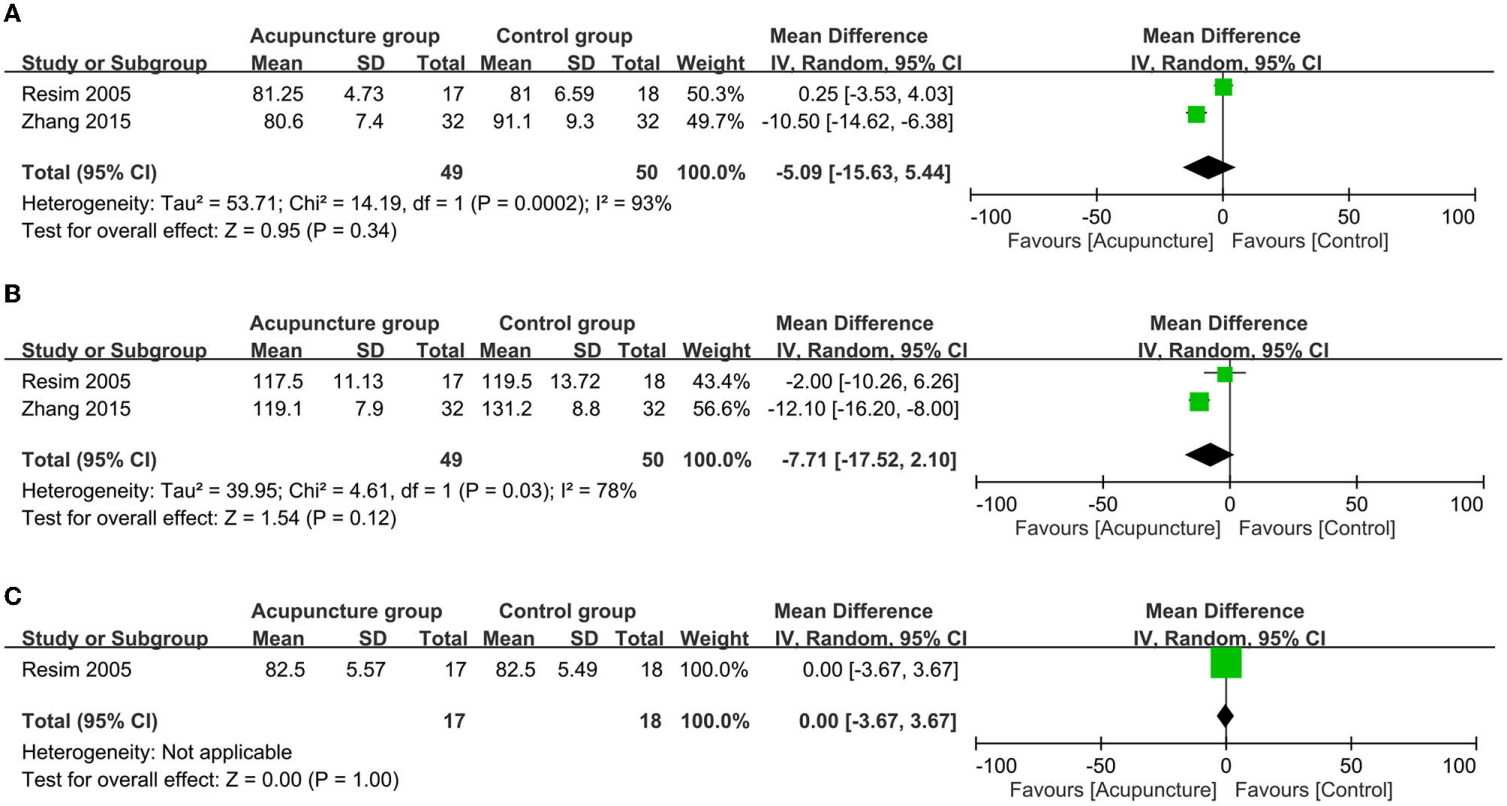
Forest plot comparing the **(A)** heart rate; **(B)** systolic blood pressure and **(C)** diastolic blood pressure during extracorporeal shock wave lithotripsy (ESWL) between acupuncture and control groups. CI, confidence interval; IV, inverse variance.

Regarding the primary outcome, pooled results indicated a better response rate associated with acupuncture compared to usual care (RR = 1.17; 95% CI: 1.06 to 1.3; *p* = 0.003; *I*^2^ = 71%; seven trials, 832 patients; Figure 3) with consistent findings on sensitivity analysis. For secondary outcomes, there was a significantly lower pain score in the acupuncture group than that in the control group (MD = −1.91 points, 94% CI: −3.53 to −0.28, *p* = 0.02, *I*^2^ = 96%, four trials, 258 patients; Figure 4A) with inconsistent findings on sensitivity analysis. Despite no difference in the duration of ESWL in both groups (MD = 0.02 min, 95% CI: −1.53 to 1.57, *p* = 0.98, *I*^2^ = 0, three trials, 141 patients, sensitivity analysis: consistent; Figure 4B), the acupuncture group exhibited a non-significantly higher stone-free rate compared to that in the control group (RR = 1.11, 95% CI: 1–1.25, *p* = 0.06, *I*^2^ = 52%, six trials, 498 patients; Figure 4C). Sensitivity analysis showed a statistically significant higher stone-free rate in the acupuncture group than that in the control group when two studies (30, 38) were removed one at a time. The hemodynamic changes during ESWL in both groups are demonstrated in Figure 5, suggesting no significant difference with or without acupuncture.

**Figure 6:**
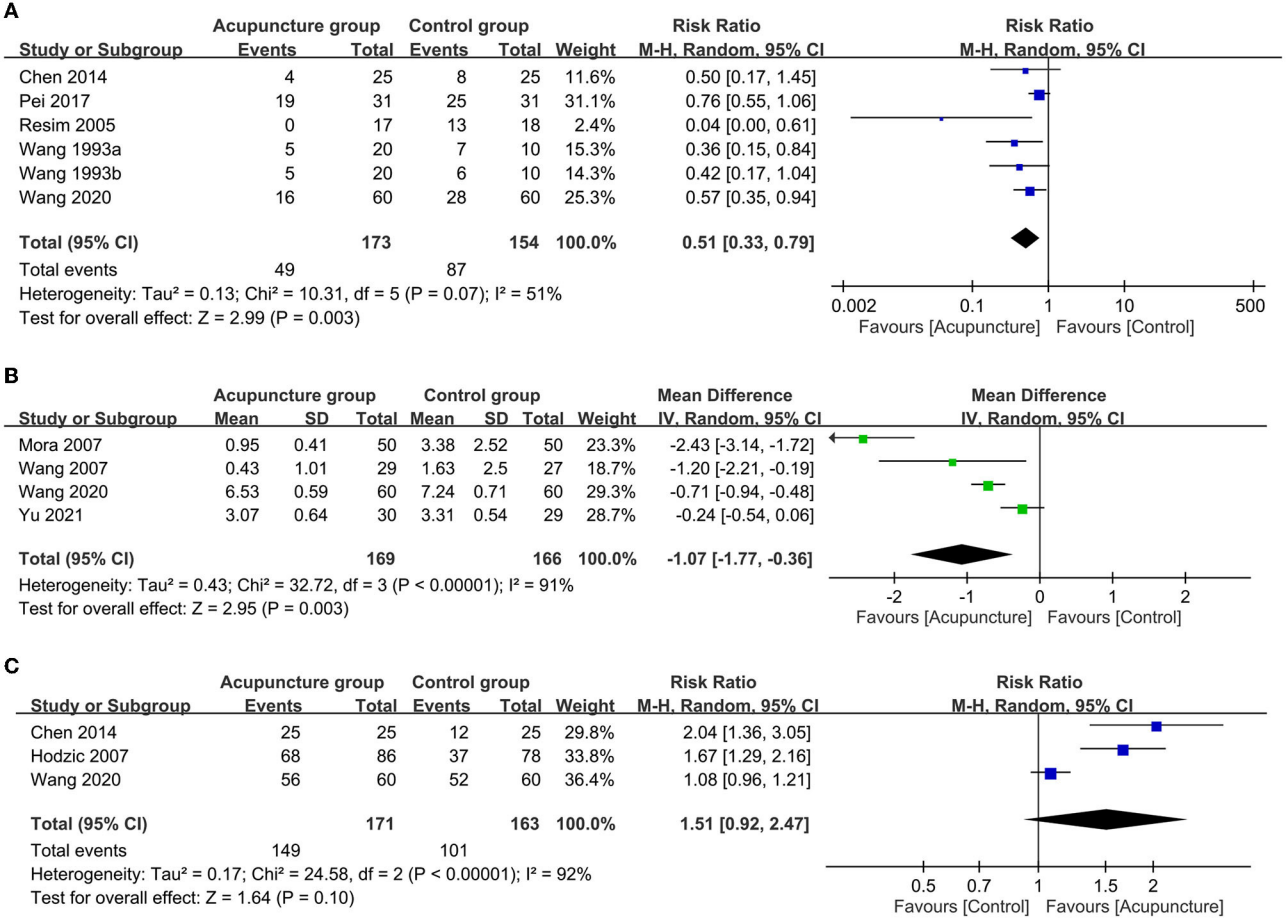
Forest plot comparing the **(A)** risk of adverse events; **(B)** post-procedural pain score and **(C)** satisfaction rate between acupuncture and control groups. RR, risk ratio; M-H, Mantel–Haenszel; CI, confidence interval.

Following ESWL, the acupuncture group showed a reduced risk of adverse events (RR = 0.51, 95% CI: 0.33 to 0.79, *p* = 0.003, *I*^2^ = 51%; five trials, 327 patients; sensitivity analysis: consistent; Figure 6A) and a lower pain score (MD = −1.07, 95% CI: −1.77 to −0.36, *p* = 0.003, *I*^2^ = 91%; four trials, 335 patients; sensitivity analysis: inconsistent; Figure 6B) compared to those in the control group. There was a non-significantly higher satisfaction rate in patients receiving acupuncture compared to that in those without (RR = 1.51, 95% CI: 0.92 to 2.47, *p* = 0.1, *I*^2^ = 92%, three trials, 334 patients; Figure 6C). Sensitivity analysis revealed a significantly higher satisfaction rate in the acupuncture group compared to the control group when the one study (34) was removed.

### 3.3. Certainty of evidence

Analysis of the quality of evidence for outcome measures based on the GRADE system demonstrated a low level of evidence in most outcomes because of downgrading due to a high degree of inconsistency and imprecision, except for diastolic blood pressure, risk of adverse events, and duration of ESWL that were all graded as high (Supplementary Table 2).

## 4. Discussion

The current meta-analysis demonstrated the effectiveness of acupuncture not only during ESWL as reflected by a higher percentage of participants experiencing effective pain relief and a reduced ESWL-related pain severity compared to those in the controls, but also after the procedure when a lower postoperative pain score and a higher stone-free rate were noted despite a lack of significant difference compared to the control group in the latter. Although there was no difference in the duration of ESWL and hemodynamic changes between the two groups, our results showed a lower risk of adverse events associated with the acupuncture approach and a non-significantly higher satisfaction rate in patients who received acupuncture.

Despite the established role of extracorporeal shock wave lithotripsy (ESWL) as an effective therapeutic approach to lithotripsy, sedation and analgesics measures are required to alleviate the considerable pain triggered by the energy dissipated as the electrical (magnetic) shock waves penetrate different tissue densities of the body (6). For pain relief in this setting, previous studies have reported the use of opiate analgesics (39), patient-controlled analgesia (PCA) (40), general anesthesia (41), epidural injection (42), local anesthesia (43), and oral analgesics. Nevertheless, drug addiction and other side effects related to conventional analgesics such as dizziness, somnolence, respiratory depression, nausea, vomiting (44), and even delayed hospital discharge (45, 46) remain an important clinical concern. In contrast to medication-induced analgesia, previous studies have explored the analgesic efficacy of the other means such as acupuncture-assisted anesthesia (AAA) (47) (including electroacupuncture, auricular acupuncture, acupuncture point injection and acupressure), transcutaneous electrical nerve stimulation (TENS) (48), massage (49), relaxation therapy, hypnosis, aromatherapy, music, and audiovisual distraction (50). One recent large-scale systematic review including 23 RCTs and 2,439 participants evaluated the efficacy of complementary therapies (i.e., music, acupuncture, acupressure, TENS, and audiovisual transfer) for pain relief in patients receiving ESWL (51). Although that study also systematically reviewed five RCTs focusing on acupuncture and related techniques in patients undergoing ESWL and described their efficacy for reducing pain score and anxiety in this patient population (51), no pooled evidence was generated to support their findings. The analgesic efficacy of acupuncture was also demonstrated in previous studies that showed a decrease in sedative and painkiller requirements during lithotripsy associated with the application of acupuncture (21, 22).

The main finding of the current meta-analysis was the analgesic efficacy of acupuncture as demonstrated by a higher percentage of participants achieving effective pain relief and a reduced ESWL-related pain severity compared to those in the control groups. Consistent with our findings, other systematic reviews have supported the promising outcomes of conventional acupuncture and related methods (e.g., auricular acupuncture, electroacupuncture, or acupoint electrical stimulation) in postoperative pain management (52) among patients undergoing back surgery (53), cardiac surgery (54), total knee replacement (55), and laparoscopic surgery (56). Therefore, our results suggested that acupuncture may be an effective analgesic strategy for patients receiving ESWL.

This study presented a significantly lower pain score in the acupuncture group than that in the control group (MD = −1.91 points, 94% CI: −3.53 to −0.28). Five of the included studies used electroacupuncture for pain relief. The analgesic mechanism of electroacupuncture may be attributed to the activation of different endogenous opioid peptides and their receptors through different frequencies of electroacupuncture stimulation (57). A previous experimental study suggested that the analgesic effect of low frequency (2 Hz) electroacupuncture stimulation in the rat spinal cord may be mediated by met-enkephalin and dynorphin acting simultaneously on the μ-, δ-, and κ*-*receptors, while that of high frequency (l00 Hz) electroacupuncture stimulation is mediated by dynorphin acting on the κ-receptor (58). It has been proposed that the release of opioid substances may produce an endogenous enkephalin-like inhibition on the projection neuron in the dorsal horn, which in turn suppresses the transmission of nociception (59).

Postoperative discomfort experienced during ESWL originates from superficial and deep somatic pain as well as visceral pain. For pain relief, previous studies have suggested the use of topical anesthetic creams such as EMLA (6), which can penetrate the skin to a depth of 4 mm. However, the analgesic effect of topical anesthetics did not reduce the need for analgesia as shown in previous RCT (60) and meta-analysis (61). The result may be explained by the inadequacy of topical anesthesia to alleviate both deep somatic pain and visceral pain (62). In contrast, previous review articles (14, 63) and meta-analysis (43) have demonstrated an effective pain relief in patients undergoing ESWL despite a similar depth of skin manipulation, suggesting the involvement of other analgesic mechanisms. The current study incorporated six conventional acupuncture studies (20, 30, 31, 33–36). Compared to other acupuncture-related techniques, conventional acupuncture induces a characteristic “grabbing needle” biomechanics known as the “de qi” reaction (64, 65). So far, de qi has been proven crucial to the effectiveness of acupuncture anesthesia (66, 67). The stronger the gripping force, the more it enhances the antinociceptive activity (68). It partially supports the theory that mechanical signals are transmitted through the connective tissue during manipulation.

For the current meta-analysis, the definition of adverse events was according to that of individual studies. Among the 13 included studies, five including six datasets provided information about the definitions and incidence of adverse events (Figure 6A) that included nausea/vomiting (23, 30, 38), dizziness (23, 30, 38), palpitation (30), orthostatic hypotension (38), renal colic (30, 33, 34), and gross hematuria (33). Of the six datasets, four adopted opioids (23, 30, 38) and two used no medication (33, 34) in their control groups. Although opioids have been shown to be effective against ESWL-related pain (69), opioid-derived analgesics are known to be associated with side effects such as nausea, vomiting, dizziness, and pruritus (70, 71). Therefore, our pooled result showing a lower risk of adverse events in the acupuncture group than that in the controls was consistent with the reported risks of adverse events associated with opioid use. Moreover, the description of a higher incidence of recurrent renal colic as the major adverse event among the recruited individuals in the two studies that enrolled control subjects with no medication (33, 34) may further imply the efficacy of acupuncture for pain relief in patients receiving ESWL.

Our study had some limitations. First, because only a small number of the included studies recruited patients undergoing sham acupuncture or sham site electrical stimulation as controls, the mechanism underlying the observed acupuncture-related analgesic effect (e.g., “de qi”) could not be elucidated. Second, insufficient information about allocation concealment as well as randomization methods and blinding in the majority of studies (i.e., eight out of 13) may have affected our results. Third, the relatively small sample size of the included studies, inconsistent interventions, different sites of intervention, and variations in study design, duration of treatment, and lithotripters were all sources of heterogeneity that may obscure the significance of our findings. Fourth, of the 48 potentially eligible articles initially retrieved, up to 30 that were included in the CNKI database could not be read in full text because of the early date of publication, illegible text from poor quality of scanning, as well as publication as posters or abstracts. As a result, those questionable articles were excluded to preserve the quality of the present study. Finally, variations in disease-related inclusion criteria (i.e., site, size, shape, hardness, and severity of obstruction) may contribute to biases in outcome interpretation. More high-quality RCTs are warranted to address these issues.

In conclusion, the results of the current meta-analysis and systematic review supported the efficacy of conventional acupuncture and related techniques for pain relief during and after ESWL. The procedures were also associated with a reduced risk of adverse events despite the lack of significant beneficial effects on stone-free rate, and patient satisfaction. Further large-scale clinical studies are required to verify our findings.

## Data availability statement

The original contributions presented in the study are included in the article/Supplementary material, further inquiries can be directed to the corresponding authors.

## Author contributions

Conceptualization and literature search: H-TC, I-WC, and C-KS. Methodology: Y-CH and I-WC. Trial selection: J-RK and K-CH. Data analysis: K-CH. Data extraction and writing—original draft preparation: H-TC and K-CH. Writing—review and editing: H-TC, K-CH, and C-KS. All authors have read and agreed to the published version of the manuscript.
