# Supplementary material for: Efficacy of acupuncture for pain relief in patients receiving extracorporeal shock wave lithotripsy: a meta-analysis of randomized controlled studies
Source: Front Med (Lausanne). 2023 Jun 2;10:1114485. doi: 10.3389/fmed.2023.1114485 (PMC10272834; doi:10.3389/fmed.2023.1114485)
Supplement: Supplementary file 1 [file Table_1.DOCX]

**Supplemental table 1.** Search strategies for Medline

| 1 | ("extracorporeal shock wave lithotripsy" or " shock wave lithotripsy" or "urolithiasis" or "nephrolithiasis" or "Ureteral calculi").mp. |
| --- | --- |
| 2 | exp "Lithotripsy"/ |
| 3 | ("Acupuncture" or "Electro acupuncture" or "Laser acupuncture" or "Needle acupuncture" or "auricular acupuncture" or "manual acupuncture" or "acupoint injection" or "acupoint treatment").mp. |
| 4 | exp "Acupuncture"/ or exp "Acupuncture Therapy"/ or exp "Acupuncture, Ear"/ |
| 5 | (1 or 2) and (3 or 4) |
| 6 | 5 and (((randomized controlled trial or controlled clinical trial).pt. or randomi*ed.ab. or placebo.ab. or drug therapy.fs. or randomly.ab. or trial.ab. or groups.ab.) not (exp animals/ not humans.sh.)) |

**Supplemental table 2.** Summary of findings for the main comparison

|  | | | | | | |
| --- | --- | --- | --- | --- | --- | --- |
| Outcomes | Effect (Risk or mean) | | Relative effect (95% CI) | № of participants  (studies) | Certainty of the evidence (GRADE) | Comments |
|  | Intervention group | Control group |  |  |  |  |
| Response rate | 384/430 | 299/402 | RR 1.17 (1.06 to 1.3) | 832 (7 RCTs) | ⨁⨁◯◯ Low | a, b |
| Peri-procedural pain score | - | - | MD -1.91 (-3.53 to -0.28) | 258 (4 RCTs) | ⨁⨁◯◯ Low | a, b |
| Duration of extracorporeal shock wave lithotripsy (ESWL) | - | - | MD 0.02 (-1.53 to 1.57) | 141 (3 RCTs) | ⨁⨁⨁⨁ High | - |
| Stone-free rate | 232/259 | 183/239 | RR 1.11 (1.0 to 1.25) | 498 (6 RCTs) | ⨁⨁◯◯ Low | a, b |
| Heart rate | - | - | MD -5.09 (-15.63 to 5.44) | 99 (2 RCTs) | ⨁⨁◯◯ Low | a, b |
| Systolic blood pressure | - | - | MD -7.71 (-17.52 to 2.1) | 99 (2 RCTs) | ⨁⨁◯◯ Low | a, b |
| Diastolic blood pressure | - | - | MD 0  (-3.67 to 3.67) | 35  (1 RCTs) | ⨁⨁⨁⨁ High | - |
| Risk of adverse events | 49/173 | 87/154 | RR 0.51  (0.33 to 0.79) | 327  (5 RCTs) | ⨁⨁⨁⨁ High | - |
| Post-procedural pain score | - | - | MD -1.07  (-1.77 to -0.36) | 335  (4 RCTs) | ⨁⨁◯◯ Low | a, b |
| Satisfaction rate | 149/171 | 101/163 | RR 1.51  (0.92 to 2.47) | 334  (3 RCTs) | ⨁⨁◯◯ Low | a, b |

Comments:

^a^wide 95% CI

^b^The I square is more than 50%.

GRADE Working Group grades of evidence:
High certainty: We are very confident that the true effect lies close to that of the estimate of the effect
Moderate certainty: We are moderately confident in the effect estimate: The true effect is likely to be close to the estimate of the effect, but there is a possibility that it is substantially different
Low certainty: Our confidence in the effect estimate is limited: The true effect may be substantially different from the estimate of the effect
Very low certainty: We have very little confidence in the effect estimate: The true effect is likely to be substantially different from the estimate of effect
